# Exploring the psychological wellbeing of women with gestational diabetes mellitus (GDM): increased risk of anxiety in women requiring insulin. A Prospective Longitudinal Observational Pilot Study

**DOI:** 10.1080/21642850.2023.2170378

**Published:** 2023-01-27

**Authors:** Emma E. Fraser, Kathryn J. Ogden, Andrea Radford, Emily R. Ingram, Joanne E. Campbell, Amanda Dennis, Anne M. Corbould

**Affiliations:** a Department of Psychiatry, Austin Hospital, Heidelberg, Australia; b School of Medicine, Faculty of Health, University of Tasmania, Launceston, Australia; c John Morris Diabetes Centre, Launceston General Hospital, Launceston, Australia; d FRANZCOG, Women’s and Children’s Service, Launceston General Hospital, Launceston, Australia

**Keywords:** Gestational diabetes mellitus (GDM), depression, anxiety, Edinburgh Depression Scale (EDS), State-Trait Anxiety Inventory (STAI)

## Abstract

**Introduction:**

Gestational diabetes mellitus (GDM) complicates ∼16% of pregnancies in Australia and has significant implications for health of both mother and baby. Antenatal anxiety and depression are also associated with adverse pregnancy outcomes. The interaction between GDM and mental health in pregnancy is poorly understood. With the aim of exploring the nuanced interaction between GDM and mental health further, we investigated whether GDM treatment modality (diet versus insulin) influenced psychological wellbeing in women with GDM.

**Methods:**

Psychological wellbeing was assessed in women with GDM treated with diet (GDM-Diet, *n* = 20) or insulin (GDM-Insulin, *n* = 15) and pregnant women without GDM (non-GDM, *n* = 20) using questionnaires [Edinburgh Depression Scale (EDS), State-Trait Anxiety Inventory (STAI-6), and in women with GDM, Problem Areas in Diabetes (PAID)] at 24–34 weeks gestation and again at ∼36 weeks gestation.

**Results:**

Women in the GDM-insulin group had significantly higher levels of anxiety than the non-GDM group at both time points. Women in the GDM-Diet group had higher levels of anxiety at 24–34 weeks gestation than the non-GDM group but did not differ at ∼36 weeks gestation. Although depression scores tended to be higher in GDM-Insulin and GDM-Diet groups than in the non-GDM group at both time points, this was not statistically significant. Diabetes-related distress was similar in the GDM-Diet and GDM-Insulin groups at both time points and did not change during pregnancy. A high proportion of the GDM-Insulin group had past/current mental illness (60%).

**Conclusions:**

In this pilot study GDM was associated with differences in psychological wellbeing, specifically increased anxiety in women treated with insulin. Specialised interventions to support women with GDM should be considered, especially those requiring insulin.

**Trial registration:** Not applicable as this was a purely observational study.

## Abbreviations

GDM: Gestational diabetes mellitus
EDS: Edinburgh Depression Scale
STAI: State-Trait Anxiety Inventory
PAID: Problem areas in diabetes

## Introduction

Women’s health encompasses both physical and mental health. Pregnancy is a time of increased risk for a woman’s health in both domains. Gestational diabetes mellitus (GDM) affects ∼16% of pregnancies in (Australian Institute of Health and Welfare, 2019). Depression and anxiety are the two most common mental illnesses to occur during pregnancy (Jha et al., 2018). The prevalence of antenatal anxiety disorders is ∼15% (Dennis et al., 2017) and depression is 7–20% (Biaggi et al., 2016). GDM and mental illness are independently associated with adverse pregnancy outcomes (Crowther et al., 2005; Grigoriadis et al., 2013). Having a comorbid diagnosis of depression and GDM has been associated with higher rates of adverse pregnancy outcomes when compared to women with GDM alone (Packer et al., 2019). The association between GDM and psychological wellbeing is complex and bidirectional (Riggin, 2020). Understanding this relationship is key to identifying potential avenues for therapeutic intervention in order to optimise outcomes for women and their babies.

The incidence of GDM has risen significantly in recent years, due to increasing rates of obesity and prediabetes in women of reproductive age, and implementation of new diagnostic criteria for GDM (Nankervis et al., 2013). Optimising maternal blood glucose levels improves obstetric and neonatal outcomes (Crowther et al., 2005). GDM also has long-term implications, conveying an increased susceptibility of the child to obesity and glucose intolerance throughout their life (Malcolm, 2012) and for mothers, an increased risk of developing Type 2 diabetes (Bellamy et al., 2009; Vounzoulaki et al., 2020). With respect to mental illness, antenatal anxiety is associated with poor pregnancy outcomes including preterm birth and low birth weight (Grigoriadis et al., 2018). A diagnosis of depression in pregnancy is associated with premature delivery and decreased initiation of breastfeeding (Grigoriadis et al., 2013). It is also associated with higher rates of postnatal depression (Robertson et al., 2004), and long-term consequences for the psychological wellbeing of the child (Stein et al., 2014).

To achieve optimal glycaemic control, women with GDM undertake blood glucose monitoring and maintain a healthy lifestyle through diet and physical activity. Women who are unable to achieve target fasting or post-meal glucose levels (Nankervis et al., 2013) receive additional therapy with insulin, typically consisting of several insulin injections daily. This burden arrives at a time when women are already increasingly susceptible to developing mental illness (Biaggi et al., 2016).

To date, few studies have examined the association between psychological wellbeing and GDM. Even less is known about this relationship with respect to different GDM treatment modalities i.e. diet versus insulin. Some studies have found that women with GDM have poorer psychological wellbeing compared to pregnant women without GDM (Kozhimannil et al., 2009; Lydon et al., 2012). Others have found no association between GDM and psychological wellbeing (Crowther et al., 2005; Katon et al., 2011; Langer & Langer, 1994). Crowther et al. found that women treated for GDM reported improved quality of life during pregnancy and at 3 months post-partum compared with women not treated for GDM (Crowther et al., 2005). However, confounding factors were greater clinical support of the intervention group, and assessment of quality of life in only a subgroup of the cohort. Similarly, there is conflicting evidence regarding the psychological wellbeing of women with GDM treated with diet versus insulin. To date, the only studies to examine this association have primarily focused on depression and have only assessed women at a single timepoint during pregnancy (Dalfrà et al., 2012; Hui et al., 2014; Kozhimannil et al., 2009; Langer & Langer, 1994). Thus, the relationship between GDM and mental state throughout pregnancy remains elusive.

Our goal was twofold. Firstly, to explore how the nuanced relationship between GDM and psychological wellbeing evolved over time. Secondly, to establish if the modality of treatment (diet vs insulin) was associated with a sustained difference in psychological wellbeing. We hypothesized that while there may be an initial increase in depression and anxiety in women with GDM, especially in women requiring insulin therapy due to the greater burden of self-care associated with this regimen, by late pregnancy psychological wellbeing would improve due to increased familiarity and confidence with diabetes self-management. To our knowledge, this is the first longitudinal study examining the impact of GDM on women’s psychological wellbeing during pregnancy that explores the potentially different effects of diet versus insulin therapy.

## Materials and methods

### Patients

This pilot study was conducted in the antenatal outpatient service of the Launceston General Hospital, a regional tertiary public hospital, over a 6-month period commencing January 2014. Women eligible for inclusion were aged 18–45 years, had a singleton pregnancy and spoke English. Women with significant pregnancy complications apart from GDM were excluded i.e. hypertensive disorders, placental disorders, foetal abnormalities, pre-existing chronic medical conditions such as epilepsy, and pre-existing psychotic disorders such as schizophrenia. Three groups of participants were assessed during the course of their pregnancies: women with GDM treated with diet and exercise (GDM-Diet), women with GDM treated with insulin (GDM-Insulin) and pregnant women without GDM (non-GDM). The non-GDM group was included as a control i.e. in order to determine if any observed changes in psychological wellbeing were specific to GDM. No women were treated with metformin in this study. GDM was diagnosed with a 75-g oral glucose tolerance test (OGTT) using diagnostic glucose thresholds of fasting ≥5.1 mmol/l and/or 1-hour ≥ 10.0 mmol/l and/or 2-hour ≥ 8.5 mmol/l or by an elevated fasting glucose alone (≥5.1 mmol/l) (Nankervis et al., 2013). Women with high risk factors for GDM (including obesity, previous GDM or macrosomic baby, first degree relative with diabetes or GDM, polycystic ovary syndrome, age ≥40 years, high risk ethnicity etc.) were screened at the first antenatal visit. All other women underwent OGTT at 24–28 weeks gestation.

In accordance with local protocol, at diagnosis of GDM, women attended a group education session provided by a dietician, diabetes nurse educator, exercise physiologist and social worker. Women were advised to perform finger-prick glucose tests four times daily i.e. fasting (target ≤5.0 mmol/l) and 2 h post-meal (target ≤6.7 mmol/l). Women unable to achieve these targets commenced insulin (i.e. bedtime Protaphane, pre-meal NovoRapid) and used a simple algorithm for adjusting their insulin doses. Consistent with local protocol, metformin was not used as a first-line agent. A diabetes nurse educator was available for advice via telephone or email. Women with GDM attended multidisciplinary antenatal clinics i.e. with access to obstetrician, midwife, dietician, diabetes nurse educator and endocrinologist.

### Assessment of mental state in GDM-Diet, GDM-Insulin and non-GDM groups

**Figure 1.**
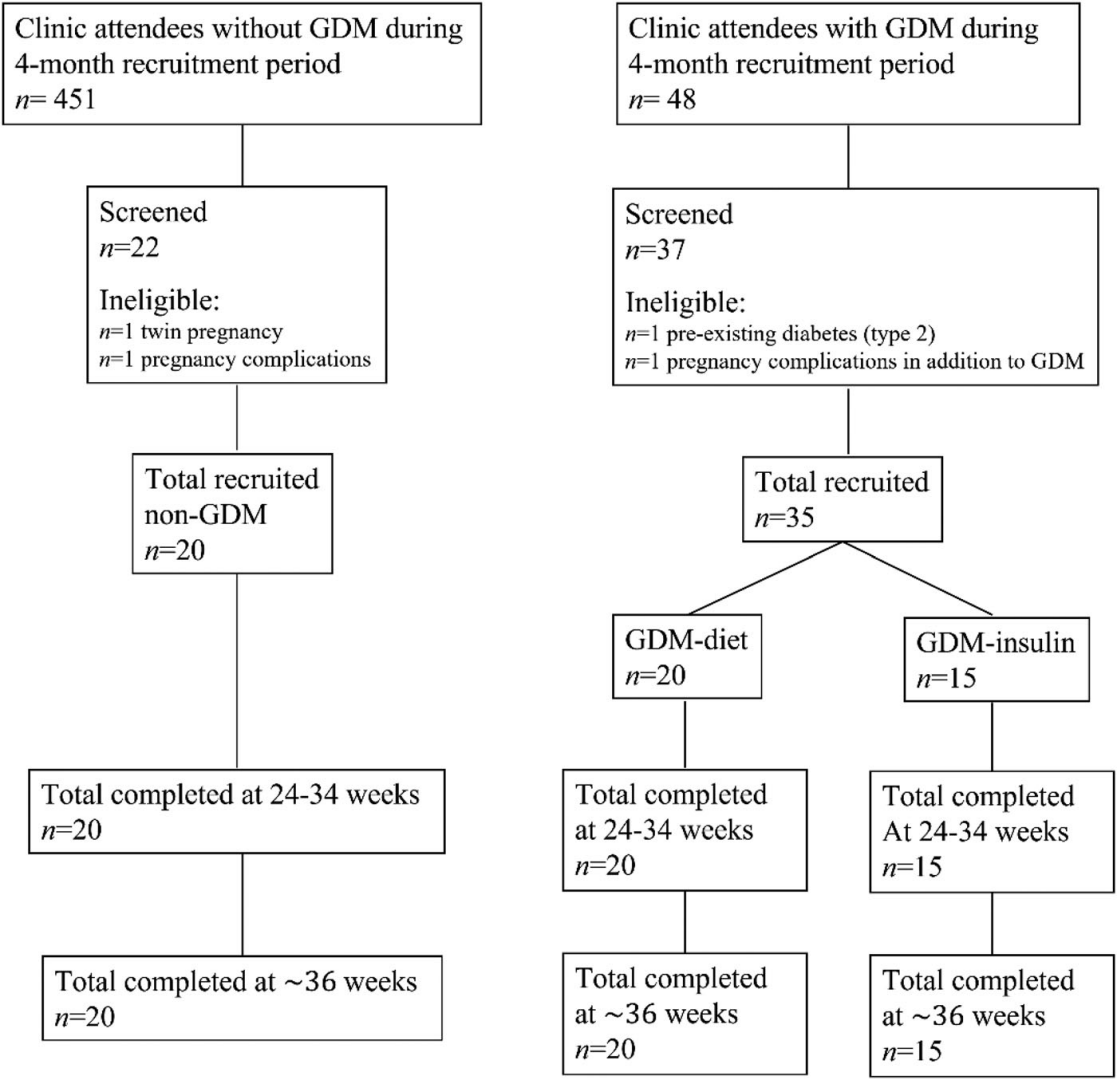
Flow chart of study recruitment.

Participants were recruited from antenatal outpatient clinics (Figure 1) and provided information regarding demographic characteristics, obstetric history, GDM risk factors, and history of significant anxiety/depression, defined as requiring prescribed medication. The specific diagnosis was not recorded. Participants completed questionnaires to evaluate psychological wellbeing at two time points, initially between 24 and 34 weeks gestation (usually at enrolment) and again at ∼36 weeks gestation. All women with GDM were initially treated with diet. Women who required insulin prior to 34 weeks gestation (GDM-Insulin group) repeated the initial questionnaires 1–2 weeks after commencing insulin. Commencement of insulin therapy after 34 weeks gestation was an exclusion criteria, likewise inability to complete the second series of questionnaires (at ∼36 weeks gestation) due to pre-term delivery, however no participants were excluded on these grounds.

### Questionnaires

The following questionnaires were used (Crowther et al., 2005; Lydon et al., 2012):

1. Speilberger State-Trait Anxiety Inventory 6 Question Short Form (STAI-6). Higher scores are associated with increased risks of anxiety. Scores below 15 are considered normal (Marteau & Bekker, 1992).
2. Edinburgh Depression Scale (EDS). Higher scores are associated with increased risk of depression. Scores above 12 indicate possible depression (Cox et al., 1987).
3. Problem Areas In Diabetes Questionnaire (PAID) was used to evaluate diabetes-related distress in women with GDM. Higher scores are indicative of increased distress. Scores above 40 indicate severe distress (Polonsky et al., 1995).

The primary outcome of the study was differences in median STAI-6, EDS and PAID scores between groups at the two time points during pregnancy.

### Statistical analysis

#### Demographic data

Independent variables included age, body mass index (BMI), parity, marital status and socioeconomic disadvantage. Socioeconomic disadvantage was based on 2006 Socio-Economic Indexes for Areas (SEIFA) Statistical Areas Level 2 (Australian Bureau of Statistics, 2006), which provides a score representing the level of disadvantage for a corresponding residential address. The mean score is 1000: lower scores are indicative of higher levels of disadvantage. Ordinal variables were compared between groups using the Mann Whitney U test. For categorical variables, groups were compared using Chi- squared or Fisher’s Exact test. The results of outcome scales were described using median and quartiles due to the ordinal non-continuous nature of the data.

#### Primary outcomes

Dependant variables included the total scores obtained from the STAI-6, EDS and PAID questionnaires. Mann Whitney U test was used to determine differences in the scores of each group at both time points. Control variables included gestation at the time of questionnaires i.e. 24–34 weeks for the first series of questionnaires and at approximately 36 weeks gestation for the second series of questionnaires. Statistical significance was accepted at a *P* value of <0.05. All data were analysed using SPSS 20 Version 22.

#### Power calculation

Pre-study power calculation based on anticipated 50% increase in EDS in women with GDM indicated sample size *n *= 15 per group. A retrospective power analysis of the study data was also performed, in the absence of actual pilot data. The number of participants included in the study was capable of detecting a 33% increase in anxiety (STAI-6 score) in the GDM diet group compared to the non-GDM group, and a 40% increase in anxiety in the GDM insulin group, with a power of 80% (Sidak method). However, the study had only a 61% power to detect a 100% increase in EDS between either GDM-Diet or GDM-Insulin and the non-GDM group.

### Ethics Statement

The study was approved by the University of Tasmania Human Research Ethics Committee (approval No. H0013537) and was performed in accordance with ethical standards of the 1964 Helsinki Declaration and later amendments. Participants provided written informed consent.

## Results

A total of 55 women completed the study (non-GDM *n* = 20; GDM-Diet *n* = 20; GDM-Insulin *n* = 15) (Table 1). No women were treated with metformin. The median SEIFA score was below 1000 in all three groups, indicating socioeconomic disadvantage. Women with GDM treated with insulin were significantly less likely to be in a married/de facto relationship than women with GDM treated with diet. A history of anxiety/depression requiring medication prior to or during pregnancy was common i.e. 35% of the non-GDM group, 15% of the GDM-Diet group and 60% of the GDM-Insulin group. Five women remained on antidepressant medication (selective serotonin reuptake inhibitors or serotonin noradrenaline reuptake inhibitors) throughout pregnancy i.e. one woman in the non-GDM group and four in the GDM-Insulin group. Thus, in comparison to the GDM-Diet group, the GDM-Insulin group had a greater proportion of women with a history of mental illness requiring medication (60% vs 15%, *P *= 0.006), more of whom continued to take this medication throughout their pregnancy (27% vs 0%, *P *= 0.026).

**Table 1.** Demographic characteristics and psychological wellbeing assessments of participants.

	Non-GDM	GDM – Diet	GDM – Insulin	*P* value
*n*	20	20	15	
Median age (IQR) – years	27.3 (22.2–30.0)	28.6 (25.8–32.6)	30.2 (29.4–35.8)	0.014 non-GDM v GDM-Ins
Median BMI (IQR) – kg/m^2^	24.8 (22.0–31.4)	24.5 (22.3–37.5)	31.0 (27.7–36.2)	0.013 non-GDM v GDM-Ins
Median SEIFA score^a^ (IQR)	976 (912–1015)	973 (901–1013)	944 (859–974)	NS
Below national average – *n* (%)	14 (70)	13 (65)	13 (87)	NS
Married or de facto – *n* (%)	15 (75)	19 (95)	10 (67)	0.028 GDM-Diet v GDM-Ins
Nulliparous – *n* (%)	4 (20)	15 (75)	4 (27)	0.001 non-GDM v GDM-Diet 0.007 GDM-Diet v GDM-Ins
Ethnicity – *n* (%)				
White	20 (100)	15 (75)	20 (100)	0.047 non-GDM v GDM
Asian		5 (25)		
Completed high school – *n* (%)	15 (75)	13 (65)	10 (66)	NS
Mental illness past/current – *n* (%)	7 (35)	3 (15)	9 (60)	0.011 GDM-Diet v GDM-Ins
Median gestation at diagnostic test for GDM (IQR) – weeks	25.1 (18.1–26.2)	26.5 (24.6–27.9)	24.0 (12.4–25.7)	0.044 non-GDM v GDM-Diet 0.020 GDM-Diet v GDM-Ins
Median gestation at 1st questionnaire (IQR) – weeks	27.8 (27.0–31.3)	29.8 (28.2–31.3)	29.8 (28.7–32.1)	NS
Median gestation at 2nd questionnaire (IQR) – weeks	36.1 (35.5–37.1)	35.3 (35.0–36.0)	35.6 (34.8–35.7)	0.040 non-GDM v GDM-Diet 0.025 non-GDM v GDM-Ins
Median STAI-6 initial (IQR)	8.0 (6.2–10.5)	10.5 (9.0–13.2)	12.0 (10.0–14.0)	0.023 non-GDM v GDM-Diet 0.005 non-GDM v GDM-Ins
Score ≥15 – *n* (%)	2 (10)	3 (15)	3 (20)	NS
Median STAI-6 ∼36 weeks (IQR)	7.5 (6.0–12.0)	10.0 (8.0–13.0)	12.0 (9.0–16.0)	0.046 non-GDM v GDM-Ins
Score ≥15 – *n* (%)	3 (15)	3 (15)	4 (27)	NS
Median EDS – initial (IQR)	2.5 (1.0–7.7)	5.0 (2.5–10.2)	6.0 (4.0–7.0)	NS
Score ≥13 – *n* (%)	2 (10)	3 (15)	1 (7)	NS
Median EDS – ∼36 weeks (IQR)	1.0 (0.0–8.0)	4.5 (3.0–8.7)	6.0 (4.0–8.0)	NS
Score ≥13 – *n* (%)	4 (20)		2 (13)	NS
Median PAID – initial (IQR)	N/A	9.0 (5.0–21.7)	9.0 (5.0–18.0)	NS
Score >40 – *n* (%)		0 (0)	1 (7)	NS
Median PAID – ∼36 weeks (IQR)	N/A	7.5 (3.2–12.5)	8.0 (3.0–17.0)	NS
Score >40 – *n* (%)		0 (0)	0 (0)	NS

^a^ Socioeconomic disadvantage was based on the 2006 Socio-Economic Indexes for Areas (SEIFA). Lower scores are indicative of higher levels of socioeconomic disadvantage.

Several differences existed in the clinical characteristics of the groups (Table 1). As expected, given the risk factors for GDM, women in the GDM-Insulin group were older than those in the non-GDM group (median age 30.2 vs. 27.3 years, *P *= 0.014) and had higher median BMI (31.0 vs 24.8 kg/m^2^, *P *= 0.013). Women in the GDM-Diet group were more likely to be nulliparous than those in the other groups. Although the initial questionnaires were completed at a similar gestation in the three groups, the median gestation at completion of the second questionnaires was slightly greater in the non-GDM group compared with the GDM-Diet and GDM-Insulin groups, a difference which is unlikely to be of relevance. Likewise, the median gestation at the time of the diagnostic OGTT was higher in the GDM-Diet group compared with other groups.

**Figure 2.**
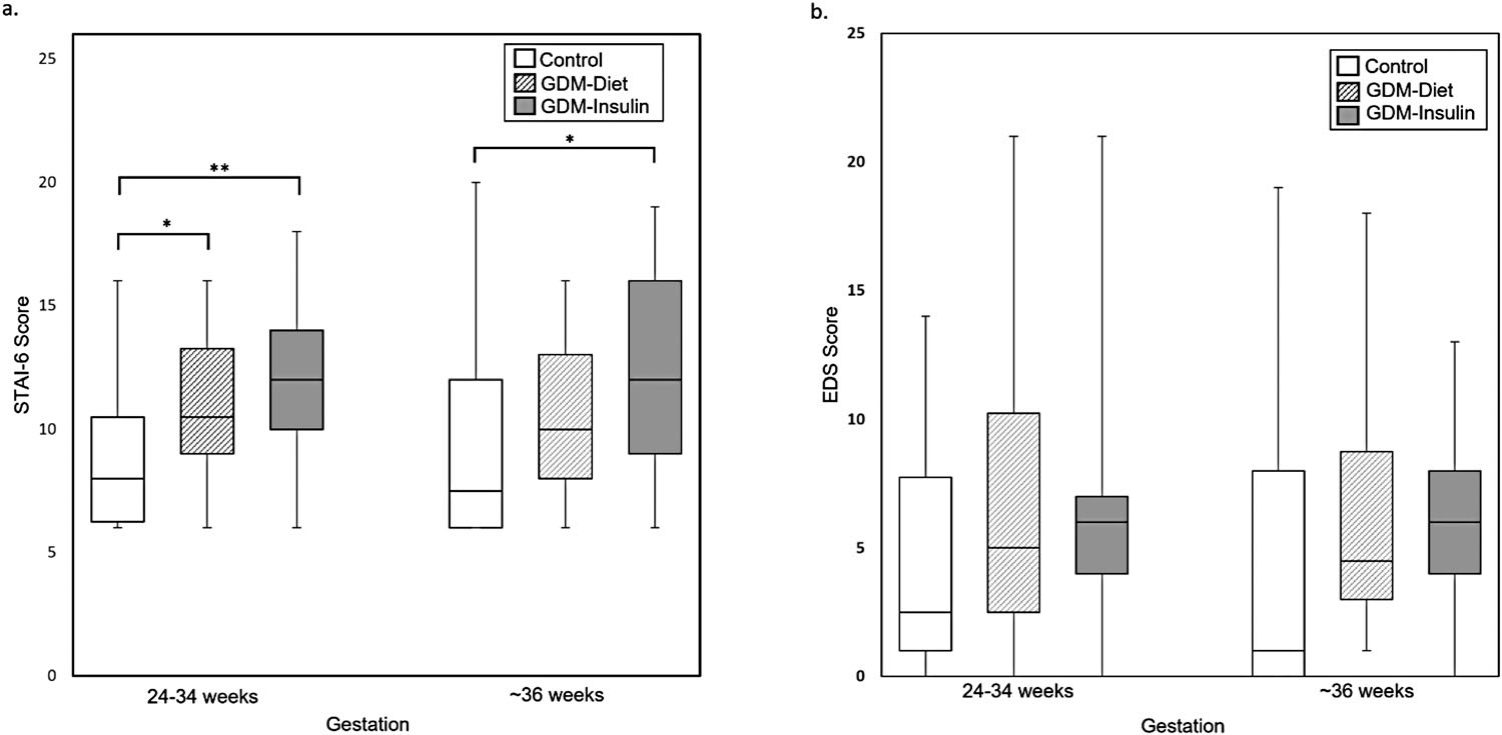
State-Trait Anxiety Inventory 6 Question Short Form (STAI-6) **(a)** and Edinburgh Depression Scale (EDS) **(b)** scores for women without GDM (non-GDM), women with GDM treated with diet (GDM-Diet) or insulin (GDM-Insulin) at two time points during pregnancy. Scores are displayed as box plots with median and interquartile range. *P < 0.05, **P < 0.01.

**Figure 3.**
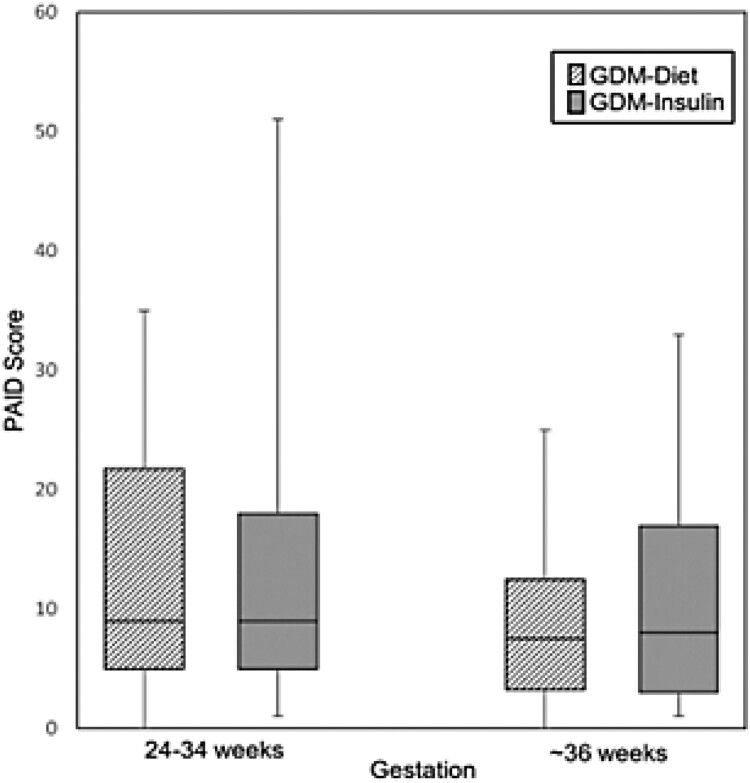
Problem Areas in Diabetes (PAID) scores in women with GDM treated with diet (GDM-Diet) or insulin (GDM-Insulin) at two time points during pregnancy. Scores are displayed as box plots with median and interquartile range.

Regarding psychological wellbeing, women with GDM treated with insulin reported significantly higher levels of anxiety at both time points compared with women in the non-GDM group (median STAI-6 score 12.0 vs 8.0, *P *= 0.005 at 24–34 weeks gestation; 12.0 vs 7.5, *P *= 0.046 (at ∼36 weeks gestation)) (Table 1, Figure 2a). At both time points, the percentage of women with STAI-6 scores ≥15 were ∼2-fold higher in the GDM-Insulin group than in the non-GDM group. In contrast, while women in the GDM-Diet group had higher levels of anxiety at 24–34 weeks gestation compared to women in the non-GDM group (median STAI-6 score 10.5 vs 8.0, *P *= 0.023), there was no significant difference at ∼36 weeks gestation. Change in STAI-6 scores did not differ between the groups. Although depression scores (EDS) tended to be higher in women with GDM (both diet and insulin-treated) than women in the non-GDM group at both time points, these differences were not statistically significant (Table 1, Figure 2(b)). Diabetes-related distress (PAID) was similar in the GDM-Diet and GDM-Insulin groups at both time points and did not change significantly between the initial and subsequent measurement (Table 1, Figure 3).

## Discussion

Our study showed that women with GDM who required treatment with insulin were at higher risk of poor psychological health during pregnancy, specifically anxiety, compared with pregnant women who did not have GDM. Contrary to our hypothesis, anxiety in women with GDM treated with insulin did not attenuate over the course of pregnancy. At ∼36 weeks gestation, 27% of women with GDM treated with insulin had scores associated with an increased risk of anxiety (STAI-6 ≥ 15). This was almost double the incidence in women with GDM treated with diet and in women who did not have GDM. Our results are consistent with two studies that showed that the psychological state of women on insulin is of greater concern than women with GDM treated with diet (Hui et al., 2014; Kozhimannil et al., 2009). Although the numbers in our study were small, the results were compelling and provide insights into avenues for further research and clinical interventions.

One of the most striking findings of this study was that women with GDM requiring insulin had higher rates of mental illness preceding pregnancy than the other groups: 60% of insulin-treated women were taking prescribed medication for anxiety/depression prior to or during pregnancy. This finding may be further evidence of the complex and bidirectional relationship between GDM and mental illness. A recent meta-analysis found that a diagnosis of depression before or during pregnancy may be associated with an increased risk of developing GDM (Arafa & Dong, 2019). In our study, this relationship appeared to hold true only for women requiring insulin. Delanerolle et al. have recently confirmed the bidirectional relationship between GDM and mental health disorders in a meta-analysis of women from Black, Asian and Minority Ethnic backgrounds (Delanerolle et al., 2021). The significant difference in pre-pregnancy mental health between insulin-treated women with GDM versus diet-treated women with GDM was a confounding factor in determining the impact of GDM on psychological wellbeing in our study. However, even though the participant numbers in our study were small, the results are relevant given that these are from a real-world clinical situation where the severity of a woman’s anxiety and implications for her pregnancy may well be unrecognised.

In women with GDM treated with diet, anxiety scores were higher than those of the women without GDM, but lower than those of women treated with insulin at both time points. Upon initial assessment, women with GDM treated with diet had significantly higher anxiety scores than women who did not have GDM, but this difference was not significant at ∼36 weeks gestation. In contrast to women with GDM treated with insulin, women with GDM treated with diet had relatively low rates of past or current mental illness. These data suggest that even in the absence of a history of mental illness or requirement for treatment with insulin, GDM has a negative impact on women’s psychological health. Alteration in eating habits potentially contributed to the initial increase in anxiety in GDM women treated with diet. However, healthy eating patterns, encompassing the typical GDM diet, have been shown to have a protective effect on mental health in pregnancy (Khan et al., 2020).

Diabetes-related stress (PAID score) did not differ in women with GDM treated with diet or insulin at either timepoint. In both groups, there was a non-significant decrease in PAID score during pregnancy, as would be expected from increasing familiarity with diabetes self-management. The PAID score exceeded the threshold for severe distress in only one woman (GDM insulin-treated) at the initial assessment and in nil women at the subsequent assessment. These results suggest that the increased risk of anxiety in women with GDM, particularly in those treated with insulin, is not entirely due to diabetes-related distress.

Our finding of sustained higher levels of anxiety in women with GDM requiring insulin has important implications for their pregnancy outcomes as anxiety has been associated with poor glycaemic control in non-pregnant populations with diabetes (Anderson et al., 2002). Despite the prevalence of anxiety disorders in pregnancy, understanding of the progression, risk factors and effects of anxiety in pregnancy is very limited compared to the extensive literature on depression (Howard et al., 2014). Women with GDM have significantly increased risk of developing antenatal depression (Lee et al., 2020; Ross et al., 2016). In our study, both groups of women with GDM reported higher depression scores compared to those in the non-GDM group, however these findings did not reach statistical significance. In Australia, women are routinely screened in pregnancy using the EDS, a scale specifically designed to detect depression. Increased emphasis on specific screening for anxiety in pregnancy may provide a practical avenue for improving outcomes for women with GDM. Further research into the relationship between antenatal anxiety and GDM should be prioritized.

The interaction between GDM and mental health is complex and the underlying mechanisms for this association are speculative. Understandably, women with GDM have concerns related to a high-risk pregnancy, their ability to manage blood glucose levels and fear of complications (Hui et al., 2014). Multiple biological and behavioural processes have been postulated to influence the association between GDM and mental health. Both GDM and depression are associated with dysregulation of the HPA axis and increased markers of inflammation (Abell et al., 2015; Harrison, 2013). Alterations in cortisol levels observed in depression have been shown to promote insulin resistance and abnormal glucose metabolism (Joseph & Golden, 2017). It is broadly accepted that the rapid hormonal changes of pregnancy create an environment primed for the development of mental illness (Altshuler et al., 2000). Depression and anxiety may also adversely affect glucose metabolism through reduction in exercise, decreased compliance with diet, medication or monitoring (Bowers et al., 2013). Whatever the mechanism, depression is also associated with poorer glycaemic control in type 1 and type 2 diabetes (Lustman & Clouse, 2005).

Socioeconomic factors are also likely to be relevant. Lydon et al found that women with GDM reported lower social support compared to pregnant women who did not have GDM (Lydon et al., 2012). Given the required lifestyle changes that women with GDM, particularly in women treated with insulin, a strong support structure would be expected to be beneficial. Although we did not explore this issue in detail, women with GDM treated with insulin in our study were significantly less likely to be in married/de facto relationships than women with GDM treated with diet. Socioeconomic disadvantage (SEIFA score) was comparable across the groups in our study, with the majority of women in each group having SEIFA scores below the national average.

The major strengths of our pilot study were that it addressed a common and highly relevant clinical issue in pregnancy and was carried out in a real-world setting. Although our study was powered to detect a moderate difference in anxiety (33%−40% increase in STAI-6 scores), a limitation was that it had a marginal likelihood of detecting a change in depression. The difference was due to greater variability in the EDS scores than in the STAI-6 scores. The small size of our study also limited our ability to conduct meaningful subgroup analyses, for example comparing outcomes from women with the lowest vs highest SEIFA scores in the 3 study groups.

In summary, GDM is a common complication of pregnancy with significant implications for the health of mother and baby. Depression and anxiety may impair the ability of affected women to achieve optimal glycaemic control. There is ongoing debate regarding how GDM impacts women’s mental health. Our study showed that women with GDM requiring treatment with insulin are at higher risk of anxiety and their psychological wellbeing did not improve during the course of pregnancy. An unexpected finding was that women with GDM requiring insulin, but not women with diet-treated GDM, had a high risk of mental illness prior to pregnancy. As a pilot study, one of the major limitations is the number of participants and larger studies are needed to validate our results. However, our findings suggest that women with GDM treated with insulin may benefit from specialised interventions. These may include screening for anxiety, early referral to psychiatry services, targeted psychology programmes and close follow-up by diabetes and obstetric clinicians. Moreover, in women with a history of mental illness, care should be taken to ensure that they undergo the recommended screening tests for GDM and have appropriate follow-up, as a diagnosis of GDM in this setting may be associated with an increased likelihood of requiring insulin. In the current environment of high workload in clinics managing diabetes in pregnancy, the ability to identify and support those women at risk of poor obstetric outcomes has the potential to significantly improve outcomes for both mother and baby.

## Data Availability

Data stored as SPSS database and is available as an SPSS or Excel document upon request.
